# Research on Confinement Effect of the Outer Steel Tube in Notched Square CFST Columns

**DOI:** 10.3390/ma15155161

**Published:** 2022-07-25

**Authors:** Biao Li, Faxing Ding, Yujie Yu, Jingke Zhang, Qiong Huang, Chenjie Gong, Haibo Wang

**Affiliations:** 1 School of Civil Engineering, Central South University, Changsha 410075, China; sddonggua@163.com (B.L.); dinfaxin@csu.edu.cn (F.D.); csdzjk@csu.edu.cn (J.Z.); gongcj@csu.edu.cn (C.G.); haibarg@163.com (H.W.); 2 China Railway Third Bureau Group Co., Ltd., Taiyuan 030001, China; 3 Hunan Airport Management Group Co., Ltd., Changsha 410137, China; 4 Shenzhen Expressway Corporation Limited, Shenzhen 518057, China; huangqiong@sz-expressway.com; 5 Hunan Tieyuan Civil Engineering Testing Co., Ltd., Changsha 410075, China

**Keywords:** concrete-filled steel tubular column, notch, finite element, compressive strength, confinement coefficient

## Abstract

The outer steel tube in a concrete-filled steel tubular (CFST) column confines the core concrete and improves the compressive strength of the core concrete. When there is a notch damage in the tube, the confinement effect may be affected. The confinement effects of the notched steel tube in rectangular CFST columns were systematically investigated by using numerical approaches. Refined three-dimensional finite element models with advanced concrete constitutive relations were established. With the verified finite element modeling method, full-sized square CFST columns with horizontal, vertical, or diagonal notches at different locations of the steel tube were simulated. Stress distributions and deformation modes of the steel tube and core concrete were analyzed. Columns with a horizontal notch at the plate center location displayed a higher axial strength reduction than those with vertical notches. A parametric study was performed to investigate the influences of concrete strengths, steel strengths, steel ratios, notch length to column width ratios, and notch angles on the compressive strengths of the rectangular CFST columns. A practical design formula was proposed based on the obtained results. The proposed formula could effectively predict the influences of different notches on the confinement effect in the notched CFST columns.

## 1. Introduction

Concrete-filled steel tube (CFST) columns have been widely used in heavy-duty structures or underground structures in recent decades, such as high-speed railway passenger stations, subways, and other underground structures [1,2,3]. The composition effect between the steel tube and infilled concrete in CFST columns enhances the axial strength and deformation performances. Compared to other composite columns with FRP tubes or steel-reinforced concrete, the CFST column presents more efficient strength elevation and better ductility [4]. Comprehensive experimental and theoretical research has been conducted on CFST columns [5,6,7,8,9,10,11,12,13,14,15,16,17]. Available studies are primarily around new CFST columns with intact steel tubes. However, the outer steel tube may experience local damage under environmental or human actions, such as erosion and regional cuttings. Local notches will be formed; then, the mechanical properties of steel tubes and CFST columns will change. The effects of the tube notches or regional damage have received some attention, and many exploring studies have been performed by researchers, e.g., Vissarion and Manolis [18], Nia et al. [19], Kabir et al. [20], Han et al. [21,22] and Gao et al. [23]. Zhu et al. [24] carried out axial compression tests on 12 circular CFST columns with circumferential or longitudinal notches at the mid-height locations of the external tubes. The presence of notches could reduce the constraining effect of the external tube on the infilled concrete. Based on the test data, the relations between the notch coefficients and the axial capacity of CFST columns under different aspect ratio conditions were proposed. Yu et al. [25] and Chen et al. [26] carried out experimental studies on the axial compression performance of circular CFST columns with different notches. The effects of notch damage on the stiffnesses, bearing capacities, and ductility properties of CFST columns were explored. However, the analyzed notches were small in the study, and the obtained effects on the axial-bearing capacities were not obvious. Chang et al. [9] conducted axial compression tests on 15 notched circular CFST columns to investigate the influence of notch lengths and orientations on the axial-bearing capacity. The compressive capacity calculation formulas of the notched circular CFST columns were then proposed.

In square CFST columns, the square steel tube has different constraining effects on the infill concrete when comparing to circular tubes. Chen et al. [26] carried out axial compressive tests on nine groups of square CFST columns with round holes. The effects of hole damage rates under different concrete strengths and slenderness ratio conditions were studied; then, axial-strength-prediction formulas for the notched square CFST columns were compared. Ding et al. [27] performed 11 groups of axial compression tests on square CFST columns with rectangular notches. The involved notches were horizontal or vertical types that were located at the center or corner locations in steel tubes to explore the impacts of notch length, orientation and location on the ductility and axial strength of CFST columns. A two-parameter formula was then proposed. Guo et al. [15] conducted 15 square CFST columns with different notch lengths, widths, thicknesses, directions, and orientations. A further parametric study was performed on notched CFST columns with different steel ratios, steel strengths, and concrete strengths using the finite element method. Based on the test data and finite element results, improved calculation formulas which can consider the strength contribution of core concrete and the strength reduction effect of the notches were studied.

Although many studies have been performed on notched square CFST columns, there is still confusion about the confinement mechanisms of notched tubes, and efficient strength calculation methods of notched rectangular CFST columns were still limited. The available studies were primarily experimental investigations based on specific notches, and then proposed formulas were often lacking in universality [28]. Previous studies mainly focused on the degradation effects of notches on column strengths. The effects of different notches on the composition action in rectangular CFST columns were often ignored. Furthermore, available formulas for the strength calculations of notched columns were often too complicated and were efficient for columns with limited certain notch types.

In view of the abovementioned problems, a systematic finite element study on notched square CFST columns was performed and illustrated in this paper. The refined three-dimensional finite element (FE) models with advanced concrete and steel constitutive relations were established. With the verified FE models, the influences of notch configurations, such as orientations (vertical, diagonal, and horizontal), locations (side plate and corner locations), and notching rates (the ratio of notch length to column dimension) on the axial compressive strengths of CFST columns with different construction conditions (concrete strength, steel strength, and steel ratio), were analyzed. Based on the large quantity of experimental and numerical data, an axial-strength calculation formula that can consider the confinement effect of notched square steel tubes in rectangular CFST columns was proposed.

## 2. Finite Element Model and Verification

### 2.1. Finite Element Model

**Figure 1:**
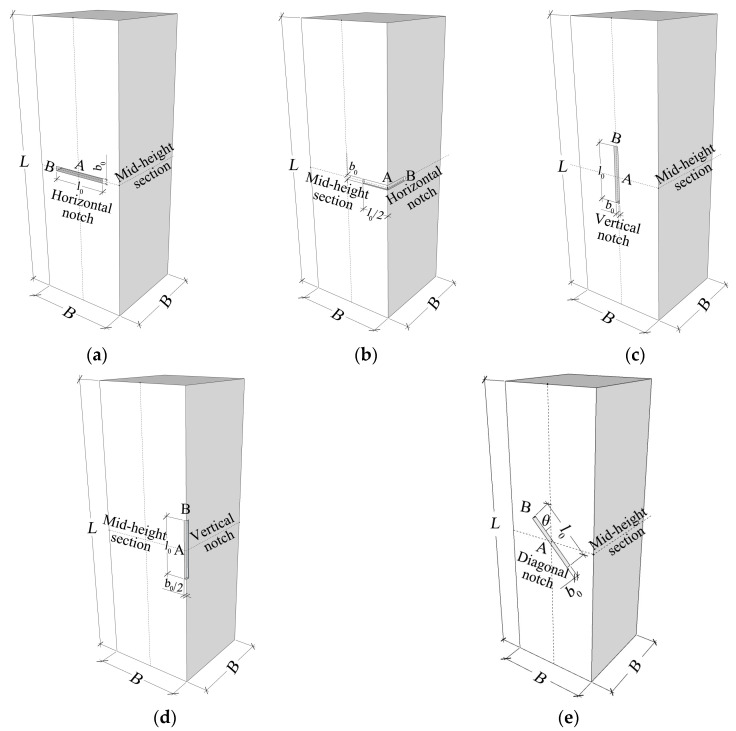
Schematic diagrams of different notches in the square CFST columns. (**a**) Horizontal notch on side plate; (**b**) Horizontal notch at the corner; (**c**) Vertical notch on side plate; (**d**) Vertical notch at the corner; (**e**) Diagonal notch on side plate.

Firstly, the refined finite element models of the notched square CFST columns were established. In the experimental study by Ding et al. [27] and Guo et al. [15], axial compressive tests were performed on a series of notched square CFST columns. The included five notch modes are illustrated in Figure 1, which included the horizontal strip notch or vertical strip notch at the side-plate center or at the corner, and the diagonal strip notch at the center location of the sidewall. To better indicate the notch configurations, dimensional parameters were defined in Figure 1, where *l*_0_ and *b*_0_ represent the notch length and width, *θ* gives the inclined angle, and *B* represents the tube width. *A* represents the center location of the notch, and *C* gives the end location of the notch. Therefore, the notch length to column width ratio *β* = *l*_0_/*B* can be obtained.

**Figure 2:**
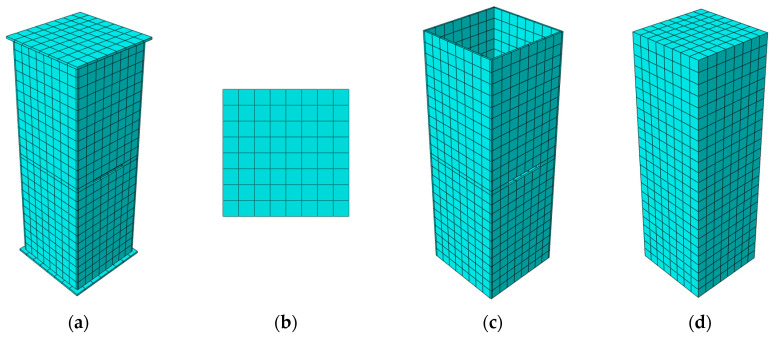
Established FE model and the meshing conditions. (**a**) FE model; (**b**) Loading plate; (**c**) Steel tube; (**d**) Core concrete.

The notched square CFST columns were modeled with the nonlinear finite element software ABAQUS 6.14 [29], and the established models are displayed in Figure 2. The steel tube, core concrete, and loading plate were all simulated with eight-node, three-dimensional solid C3D8R elements with the hourglass control. The models were structurally meshed in the hexahedral mode with a meshing size of 50 mm. The surface-to-surface contact interactions were established between the outer tube and core concrete. The inner surfaces of the steel tube were settled as main surfaces, and outer surfaces of the core concrete were slave surfaces. The normal interactions were governed with the “hard” contact, and the tangential friction was calculated with the penalty function with a Coulomb friction of 0.5. Loading plates were included and were tie-connected to the column ends to ease the axial-load application. Loading plates were modelled as rigid plates with a high elastic modulus of 1 × 10^11^ MPa and a small Poisson ratio of 1 × 10^−7^. The bottom loading plate was fixed at displacement and rotational freedoms. The axial compressive displacement load was applied onto the top loading plate and then simultaneously transferred to the core concrete and outer tube, simulating the real load-bearing conditions in CFST columns.

The core concrete adopted the advanced concrete damage plasticity constitutive model that was proposed by Ding et al. [30]. The concrete model was described with triaxial constitutive relations described by the following equations.

(1) $$y=\left\{ \begin{array}{ll} \frac{A_{1}x+(B_{1}-1)x^{2}}{1+(A_{1}-2)x+B_{1}x^{2}} & x\leq 1\\ \frac{x}{\alpha_{1}(x-1{)}^{2}+x} & x>1 \end{array}\right.$$

**Figure 3:**
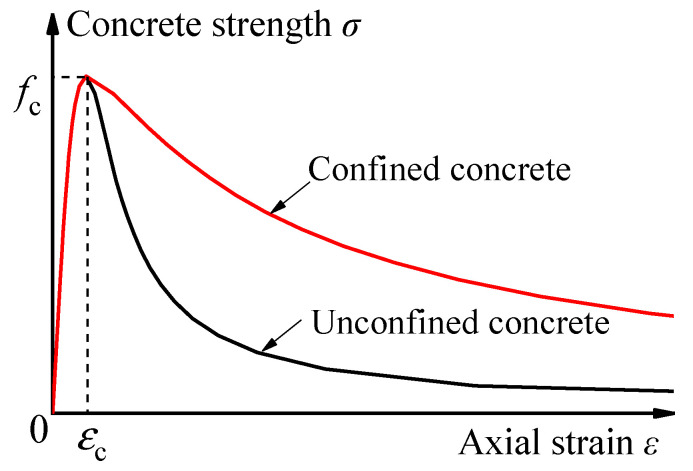
Schematic diagram of the triaxial constitutive relation of concrete.

For the restrained core concrete in the compressed CFST columns, *y* = *σ*/*f*_c_ and *x* = *ε*/*ε*_c_, where *σ* and *ε* signify the stress and strain. *f*_c_ is the axial compressive strength and can be calculated by the cubic compressive strength of concrete *f*_cu_ as *f*_c_ = 0.4*f*_cu_^7/6^. *ε*_c_ is the strain that corresponds to the compression peak stress, and can be calculated by *ε*_c_ = 291 *f*_cu_^7/15^ × 10^−6^. The schematic diagram of the triaxial constitutive relation of concrete is shown in Figure 3. *A*_1_ and *B*_1_ are the parameters for the strength-hardening stage (*x* ≤ 1), and can be calculated by *A*_1_ = 6.9*f*_cu_^−11/30^ and *B*_1_ = 1.6(*A*_1_ − 1)^2^. *α*_1_ is the parameter at the strength-descending stage, and is equal to 0.15 in this study. The Poisson’s ratio of concrete was 0.2 in those FE models.

In ABAQUS, the concrete plasticity damage model was applied to the core concrete. The practical material strength relations were calculated through the abovementioned equations and were incorporated into the concrete plasticity damage model. Other parameters were defined in reference to the previous studies by Ding et al. [30]: The Poisson’s ratio of concrete was 0.2, and the dilatancy angle was 40° [31]. When the concrete is subjected to the biaxial isobaric stress, the strength was 1.277 times the uniaxial strength [31], and the eccentricity and viscosity coefficients were 0.1 and 0.0005, respectively.

The steel constitutive model took the multilinear kinematic relation that is described using the following equations by Ding et al. [30].

(2) $$\sigma_{1}=\left\{ \begin{array}{ll} E_{s}\varepsilon_{i} & \varepsilon_{i}\leq \varepsilon_{y}\\ f_{s} & \varepsilon_{y}<\varepsilon_{i}\leq \varepsilon_{\mathrm{st}}\\ f_{s}+E_{\mathrm{st}}(\varepsilon_{i}-\varepsilon_{\mathrm{st}}) & \varepsilon_{\mathrm{st}}<\varepsilon_{i}\leq \varepsilon_{u}\\ f_{u} & \varepsilon_{i}>\varepsilon_{u} \end{array}\right.$$

where *σ*_i_ is the equivalent plastic strength, *E*_s_ is the elastic modules, and *f*_s_ and *f*_u_ are the yield strength and ultimate strength of steel, respectively. The ultimate strength was correlated with yield strength by the following relation: $\frac{f_{u}}{235}=0.86\frac{f_{y}}{235}+0.72$. The Poisson’s ratio was 0.285. Parameters *ε*_i_, *ε*_y_, *ε*_st_, and *ε*_u_ signify the equivalent strain, the yield strain, the strain corresponding to the initiation of strength hardening, and the strain corresponding to the achievement of the ultimate plastic strength of steel. Those strains were correlated with each other by the following relations: *ε*_st_ = 0.02, $\frac{\varepsilon_{u}}{\varepsilon_{u,235}}=\frac{1}{1+0.15{\left(f_{y}/235-1\right)}^{1.85}}$, and *ε*_u,235_ = 0.12. The modules of the steel strengthening *E*_st_ = (*f*_y_ − *f*_u_)/(*ε*_u_ − *ε*_st_). 

### 2.2. Model Verification

**Figure 4:**
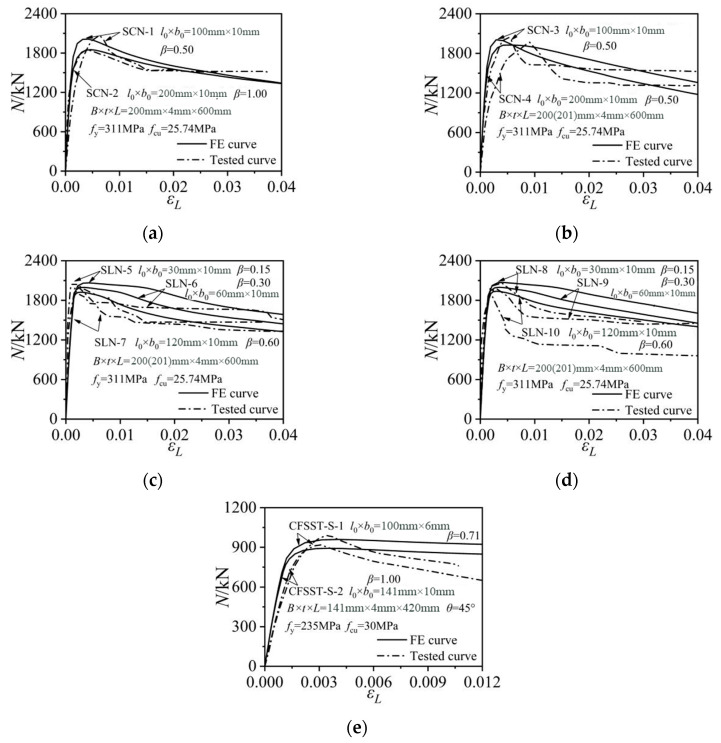
The comparisons of the finite element and test results. (**a**) Specimens SCN1~SCN2; (**b**) Specimens SCN3~SCN4; (**c**) Specimens SCN5~SCN7; (**d**) Specimens SCN8~SCN10; (**e**) Specimens CFSST-S-1~CFSST-S-2.

Figure 4 shows the comparisons of the numerical results with the test data obtained by Ding et al. [27] and Guo et al. [15]. The adopted modelling methods and the established models can effectively predict the ultimate strengths of the tested notched CFST columns. The initial stiffnesses of SCN3/4 and CFSST-S-1/2 that were obtained from the experimental tests were slightly smaller than the corresponding FE values, which might be caused by the displacement measuring deviations in practical tests. Given *N*_uc,t_ as the ultimate strength of the tested specimens, and *N*_uc,FE_ as the corresponding ultimate strength predicted by finite element models, the mean value and the discrete coefficient *N*_uc,t_/*N*_u,FE_ were 1.007 and 0.009, respectively. The established finite element models can well predict the axial strengths of the notched square CFST columns, and can then be used for the following parametric analysis.

## 3. Parameter Analysis

Based on the verified FE modeling method, full-scale square CFST columns with different notching modes were established to investigate the mechanical properties and the composite action. The studied column had a standard configuration as *L* × *B* × *t* = 1500 mm × 500 mm × 10 mm with a steel ratio *ρ* of 0.085, where *B* is the external width, *t* is the plate thickness of the steel tube, and *L* is the height of the column. The cubic compressive strength of the core concrete, *f*_cu_, was 60 MPa and the steel yield strength *f*_y_ was 345 MPa. A strip notch with a width (*b*_0_) of 20 mm was slotted at the mid-height location of the steel tube. In the parametric analysis, the notch length (*L*_0_) varied from 50 mm, 100 mm, 250 mm, 400 mm to 500 mm with the notch length to column width ratios (*β*) from 0.1, 0.2, 0.5, 0.8 to 1, respectively. The inclined angle of the strip notch varied from 0°, 30°, 45°, 60° to 90° to simulate the horizontal, diagonal, and vertical notches. In addition, the FE model of the intact square CFST column was also established and simulated for comparison. 

### 3.1. Column Strength–Axial Strain Relations

**Figure 5:**
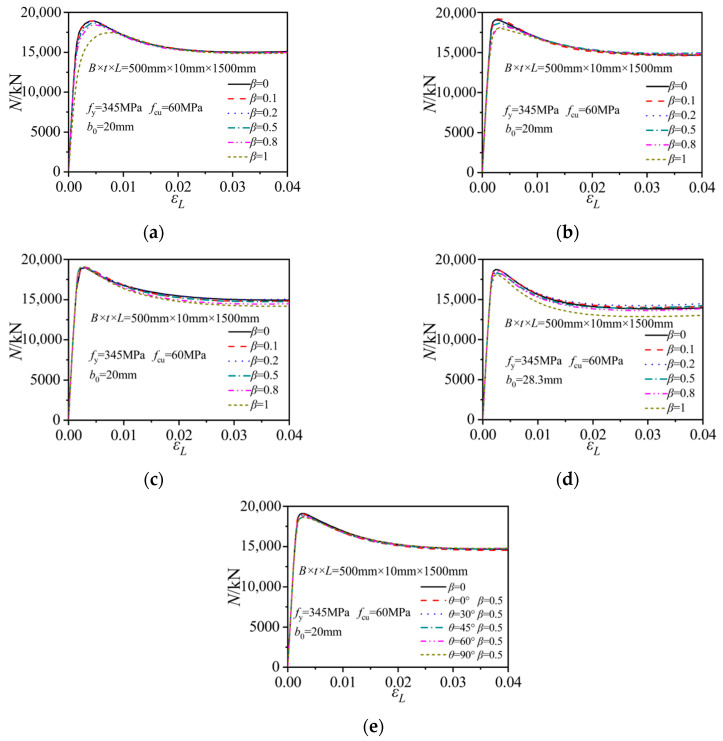
Axial strength–axial strain relations of the columns under different notching modes. (**a**) Horizontal notch on the side plate; (**b**) Horizontal notch at the corner; (**c**) Vertical notch on the side plate; (**d**) Vertical notch at the corner; (**e**) Diagonal notch on the side plate.

Figure 5 shows the axial column strength versus axial strain relations of the notched CFST columns under different notching modes. For the horizontal notches at the side-plate center of the steel tube, the initial elastic stiffness and the ultimate bearing strength of the rectangular CFST columns gradually decreased with the increase in the notch length to column width ratio *β*. When *β* reached 1, the ultimate axial strength decreased by about 7.8% compared to that of the intact column. The notch configurations exerted little effect on the residual column strength during the later loading stage with the axial strain being higher than 0.01. For the horizontal notches at the corner of the steel tube, the initial stiffness and residual strength developments were basically unaffected by the variations in *β*. The reduction effect of the notch on ultimate strength was reduced. The axial strength of the column with *β* as 1 only decreased by about 5.5% compared to that of the intact column.

For the column with a vertical notch on the side plate or at the corner, the notch length had little effect on the initial stiffnesses of the composite columns, as indicated in Figure 5c,d. The increase in the notch length merely reduced the residual strength levels. When the vertical notch was located at the side-plate center, the ultimate strength remained unaffected despite the variations in the notch lengths. When the vertical notch was positioned at the tube corner, the axial strength only suffered a slight reduction when *β* approached 1.0.

Figure 5e compares the strength developments of the diagonally notched columns with different notch inclination angles. When *θ* increased from 0° to 90°, the elastic stiffness and residual strength developments remained nearly unchanged, while the ultimate strength was slight reduced. The maximum strength of the column with a vertical strip notch (*θ* = 90°, *β* = 0.5) was about 2.3% smaller than that of the model with a horizontal notch (*θ* = 0°, *β* = 0.5). Furthermore, the square CFST column with vertical notches at the side-plate center displayed the highest axial strength, while the column with a horizontal notch on the side plate suffered the maximum stiffness and axial strength reductions.

### 3.2. Stress States of the Steel Tubes

**Figure 6:**
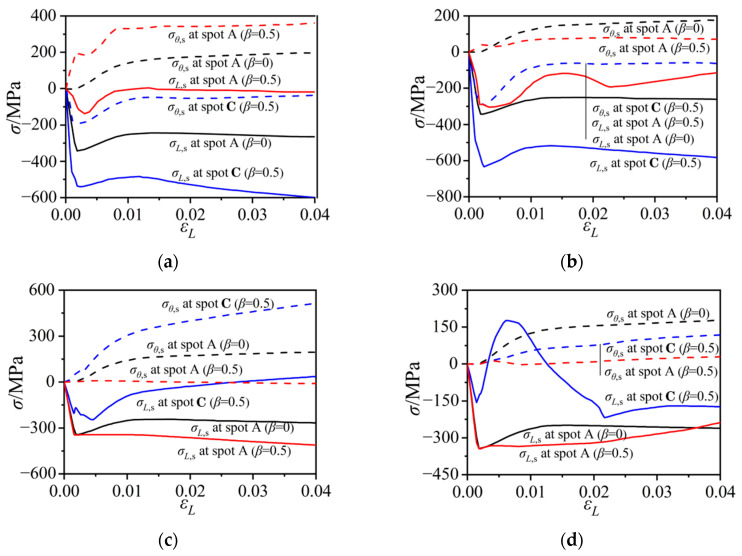
Steel stress versus the column axial-strain relations under different notching modes. (**a**) Horizontal notch on the side plate; (**b**) Horizontal notch at the corner; (**c**) Vertical notch on the side plate; (**d**) Vertical notch at the corner.

To investigate the effect of notch damage on stress states in the outer tube, nodal stresses at the midpoints of the notch length and width (spots A and B in Figure 1) were extracted and compared, as illustrated in Figure 6. The column group with 250 mm-long notches (*β* = 0.5) was selected for comparison. The tensile stresses along the longitudinal direction (*σ_L_*_,s_) and the horizontal direction (*σ_θ_*_,s_) were represented by the positive curves. In the intact CFST column (*β* = 0) (as illustrated in Figure 6a), the side plate was in a compressive state due to the axial-load bearing contribution of the steel tube, and also had a tensile stress around 150 MPa along the horizontal direction due to the confinement effect to the core concrete. However, for the column with a horizontal notch at the side-plate center, the steel plate around the notch (spot A in *β* = 0.5 case) was in the uniaxial tensile mode along the horizontal direction, and the axial stress along the longitudinal direction was small. The axial compressive stress at spot B of the 250 mm notch increased quickly during the initial loading stage and then remained at a high level, exceeding the steel yield strength, while the horizontal stress at spot B increased during the initial compression stage, and then reduced to a low level. The horizontal notch intercepted the axial load transfer along the damaged side plate, maintaining only the confinement effect to the core concrete. The stress flow in the steel tube was obstructed and stress concentrations were formed at two ends of the notch, resulting in the complex stress states at spot B.

In the intact CFST column, the axial and horizontal stresses at the corner location were similar to those at the side-plate center, which indicated an average stress state across the column section. For the column with a horizontal notch at the corner, the axial stress at the corner (spot A in *β* = 0.5 case) presented similar compressive stress developments during the initial loading stage to that of the intact column, while the horizontal stress at spot A remained at a low level. The notch ends (spot B in *β* = 0.5 case) were in a bidirectional compressive state during the initial loading stage. The axial stress at spot B remained around −600 MPa during the later loading stage, while the horizontal stress reduced to a low level when the axial column strain exceeded 0.01.

Figure 6c,d compare the stress developments of the vertically notched columns. For the column with a vertical notch at the side-plate center, the axial stress at two sides of the notch was in uniaxial compressive states with the horizontal stress being equal to 0. At two ends of the notch, the steel tube was longitudinally in compression until the achievement of the maximum column strength. The axial compressive stress then decreased to 0 in the later loading stage. The horizontal stress at spot B was positive (in tension) and the stress level continued to increase during the entire loading process.

For the column with a vertical notch at the corner of the tube, the axial stresses around two sides of the notch were in compression, and the stress development was similar to that in the intact column. The confinement effect of the steel tube to the core concrete was intercepted by the notch, leading to nearly 0 horizonal stress at two sides of the notch. The notch end (spot B) experienced dramatic stress variations due to the local buckling deformation. Therefore, the vertical notch reduced the confinement effect of the outer tube to the core concrete.

### 3.3. Concrete Stresses

**Figure 7:**
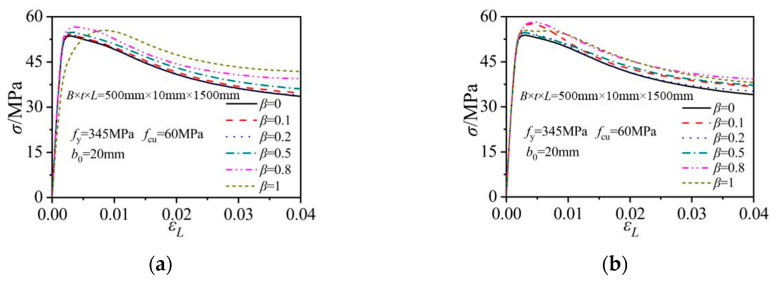

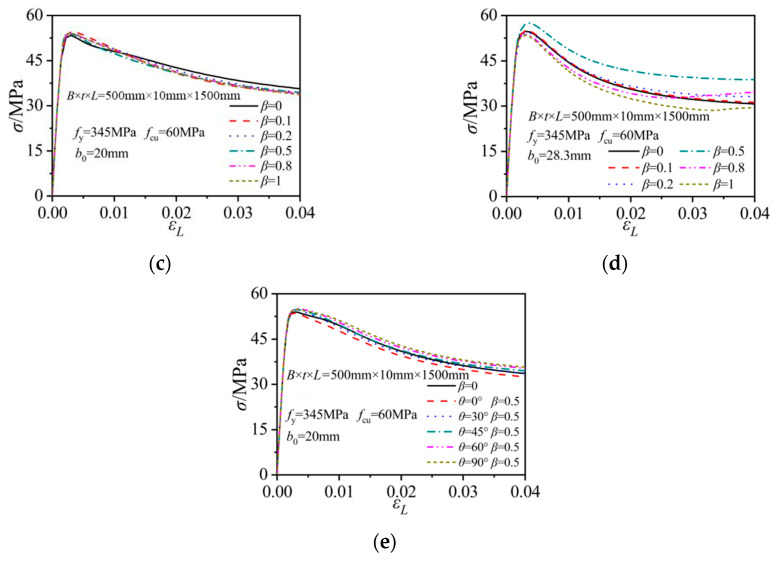
Average concrete stress versus axial column strain relations under different notching modes. (**a**) Horizontal notch on the side plate; (**b**) Horizontal notch at the corner; (**c**) Vertical notch on the side plate; (**d**) Vertical notch at the corner; (**e**) Sloping notch on the side plate.

The behavior of the core concrete was described by the average axial concrete stress at the mid-height section. The obtained concrete stress versus axial column strain relations were obtained and are compared in Figure 7. In the columns with horizontal notches (at the steel side-plate center and at the corner location), the peak axial stress in the core concrete increased when comparing to that in the corresponding intact column. The horizontal notch reduced the axial-load-bearing portion of the steel tube, therefore increasing the load share and stress levels in the core concrete. The concrete stress elevation increased with the increase in *β*. For the column with a 500 mm-long notch at the side-plate center (*β* = 1 in Figure 7a), the core concrete displayed an earlier nonlinear strength increase but a latter achievement of peak strength when comparing to the intact column. When the horizontal notch was located on the steel tube, the increases in the notch lengths had little effect on the initial increasing rate of the concrete stress.

In the columns with vertical notches, the notch length had little effect on the initial increasing rate of the concrete stresses. The peak concrete stress in the columns with the vertical notch on the side plate remained nearly unchanged with the variations in *β*. When the vertical notch was located at the tube corner, the notch length affected the peak concrete stress levels and the following concrete stress degradation modes. The column with a 250 mm-long notch (*β* = 0.5) exhibited the maximum peak concrete stress. The peak concrete stress in the column with a 500 mm-long notch (*β* = 1) was the minimum. Compared to the horizontally notched columns, the notch length had a weaker effect on the concrete stress in the corresponding vertically notched ones.

As illustrated in Figure 7e, the notch inclination angle had only a little effect on the concrete stress when the notch was at the side-plate center. The peak concrete stress slightly increased with the increase in *θ*. Therefore, the concrete stress was the lowest in the vertically notched column and was the highest in the horizontally notched column. These concrete stress variations also indicated that the confinement effect of the outer tube was lower in the vertically notched column, when comparing to the horizontally notched one.

### 3.4. Stress Contours

**Figure 8:**
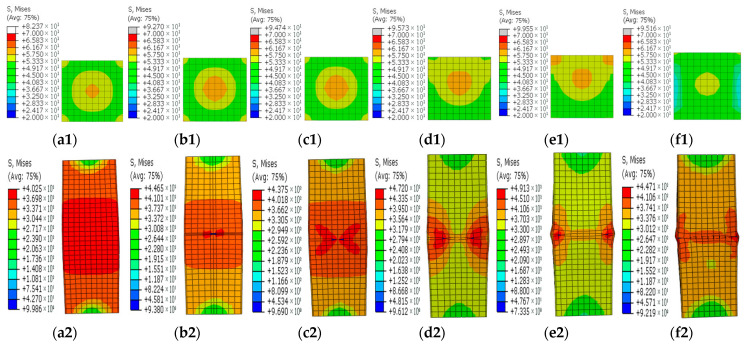
Concrete and steel stress contours in the steel tube with horizontal notches on the side steel plate for different *β* values. (**a1**–**f1**) Concrete stress contours for different *β* values; (**a2**–**f2**) Stress contours in the steel tube for different *β* values. (**a1**,**a2**) *β* = 0; (**b1**,**b2**) *β* = 0.1; (**c1**,**c2**) *β* = 0.2; (**d1**,**d2**) *β* = 0.5; (**e1**,**e2**) *β* = 0.8; (**f1**,**f2**) *β* = 1.

**Figure 9:**
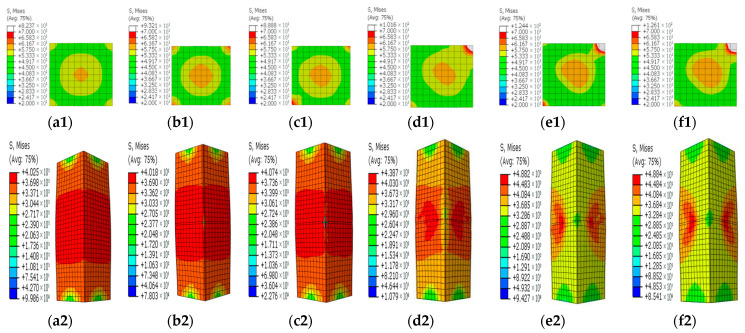
Concrete and steel stress contours in the steel tube with horizontal notches at the corner for different *β* values. (**a1**–**f1**) Concrete stress contours for different *β* values; (**a2**–**f2**) Stress contours in the steel tube for different *β* values. (**a1**,**a2**) *β* = 0; (**b1**,**b2**) *β* = 0.1; (**c1**,**c2**) *β* = 0.2; (**d1**,**d2**) *β* = 0.5; (**e1**,**e2**) *β* = 0.8; (**f1**,**f2**) *β* = 1.

**Figure 10:**
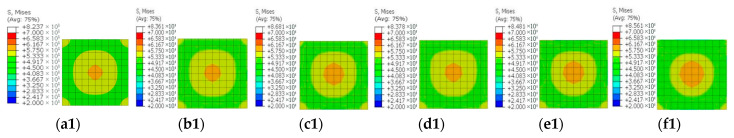

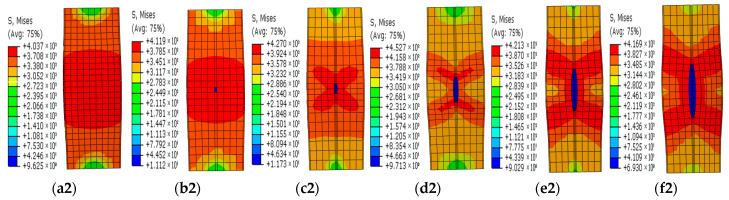
Concrete and steel stress contours in the steel tube with vertical notches on the side steel plate for different *β* values. (**a1**–**f1**) Concrete stress contours for different *β* values; (**a2**–**f2**) Stress contours in the steel tube for different *β* values. (**a1**,**a2**) *β* = 0; (**b1**,**b2**) *β* = 0.1; (**c1**,**c2**) *β* = 0.2; (**d1**,**d2**) *β* = 0.5; (**e1**,**e2**) *β* = 0.8; (**f1**,**f2**) *β* = 1.

**Figure 11:**
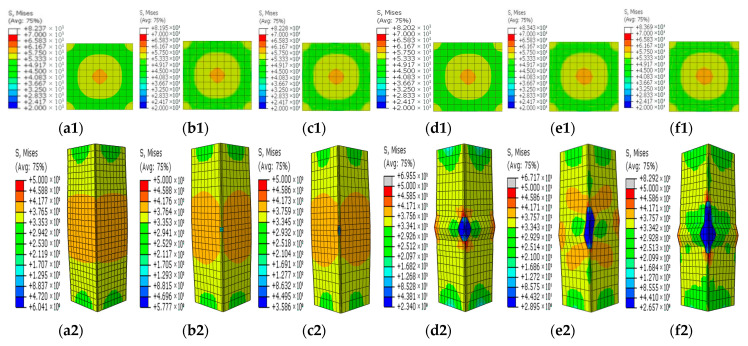
Concrete and steel stress contours in the steel tube with vertical notches at the corner for different *β* values. (**a1**–**f1**) Concrete stress contours for different *β* values; (**a2**–**f2**) Stress contours in the steel tube for different *β* values. (**a1**,**a2**) *β* = 0; (**b1**,**b2**) *β* = 0.1; (**c1**,**c2**) *β* = 0.2; (**d1**,**d2**) *β* = 0.5; (**e1**,**e2**) *β* = 0.8; (**f1**,**f2**) *β* = 1.

**Figure 12:**
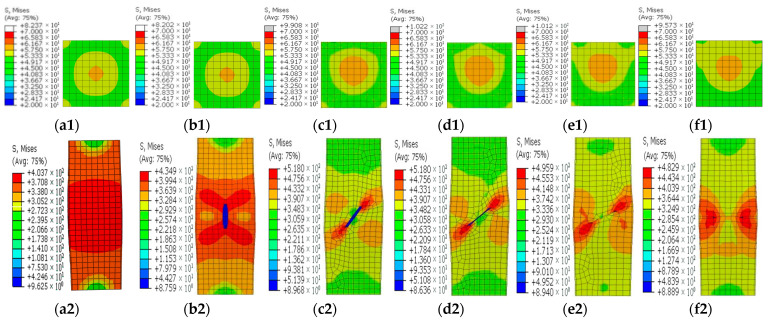
Concrete and steel stress contours in the steel tube with diagonal notches on the side steel plate for different *β* values. (**a1**–**f1**) Contours for different *β* values; (**a2**–**f2**) Stress contours in the steel tube for different *β* values. (**a1**,**a2**) *β* = 0; (**b1**,**b2**) *θ* = 0°; (**c1**,**c2**) *θ* = 30°; (**d1**,**d2**) *θ* = 45°; (**e1**,**e2**) *θ* = 60°; (**f1**,**f2**) *θ* = 90°.

Figure 8, Figure 9, Figure 10, Figure 11 and Figure 12 show the stress contours in the steel tube and core concrete at the failure state. In the columns with a horizontal notch on the side plate, the core concrete changed from the axially loaded state to the eccentric compression state with the increases in the notch lengths. The steel tube in the intact CFST column had a globally outward expansion deformation. For the column with a horizontal notch on the tube side plate, the discontinuity resulted in stress concentrations and local buckling of the tube plate at the mid-height location. Relative slips occurred between the steel plate and the core concrete around the notch. As *β* exceeded 0.5, the notch was nearly closed due to the buckling deformation of the rest tube plates and excessive slips between the steel plate and core concrete around the notch.

In the square CFST columns with vertical notches, the core concrete stayed in the axially loaded state in spite of the different notch lengths. However, with the increase in *β*, the steel tube tended to buckle outwardly with an increased level of uneven stresses around the notch. Stress concentrations occurred at two ends of the notch, and the concentration level increased in the long notch cases. When *β* was greater than 0.5, the steel-plate regions around the notch buckled outwardly. The steel plates around the notch were separated with the core concrete during the failure and later strength degradation stages.

In the columns with diagonal notches, the bearing mode of the core concrete changed from the axially loaded type to the eccentric loaded mode with the increases in the notch inclination angles. When the inclined angle of the notch was smaller than 45 degrees, the outward expansion deformation was exhibited in the tube plates around the notch. The failure stress development modes were similar to those of the vertically notched columns. When the inclined angle was larger than 45 degrees, the notch damage tended to be close, presenting a similar deformation mode to that in the horizontally notched column. Stress concentrations were observed at two notch ends.

## 4. Practical Design Formula

### 4.1. Parametric Analysis

The results in the above discussions indicated that the core concrete was in eccentric loading states in horizontally notched columns, while in the vertically notched columns, the core concrete remained in axially loaded modes, and the notch primarily influenced the confinement effect of the outer tube. In order to describe the axial strength reduction in the notched columns, an influence coefficient *SI* was introduced, which was defined as the ratio of the compressive strength of the notched columns (*N*_uc_) to the axial strength of the corresponding intact column (*N*_u_):

(3) $$SI=\frac{N_{\mathrm{uc}}}{N_{u}}$$

To explore the relation of *SI* with notch configurations and column construction parameters, an extended parametric study with 408 FE models was performed. The involved parameters were the notch length to column width ratio *β*, the concrete strength *f*_cu_, the steel strength *f*_y_, and the steel ratio *ρ*. The basic column dimensions (*B* and *L*) and the definition of *β* remained the same as those in Section 3.

**Figure 13:**
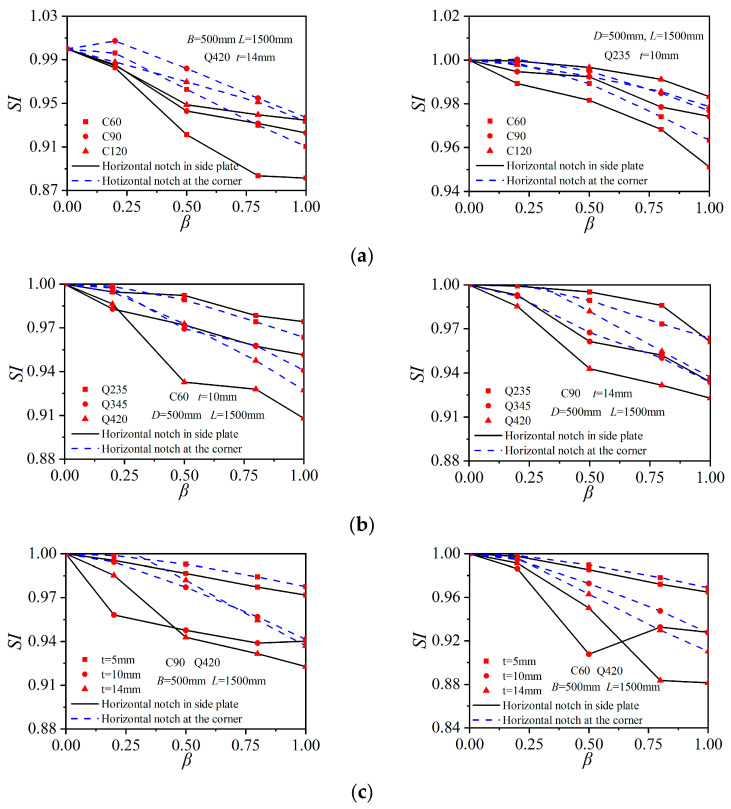
Impacts of the parameters on the ratio *SI*. (**a**) Influence of the concrete strength; (**b**) Influence of the steel strength; (**c**) Influence of the steel ratio.

Figure 13 shows the relations of different parameters versus the coefficient *SI* in the horizontally notched columns. The results indicated that the ratio *SI* got reduced with the increase in *β*, and the corresponding pattern was in accordance with the above discussions. The ratio *SI* still increased with the increase in concrete strength (other parameters remained constant), indicating a limited reduction effect of notches on the axial-bearing strength. As shown in Figure 13b, when the steel strength was elevated, the coefficient *SI* got reduced. Moreover, the side-plate horizontal notch case displayed more dramatic strength reductions in the columns with Q420 steel tubes. The coefficient *SI* primarily got reduced with the increase in the steel ratio *ρ*. When the steel plate thickness increased from 5 mm to 10 mm (corresponding to the steel ratios of 0.041 and 0.085, respectively), the ratio *SI* decreased dramatically, while when the steel plate thickness further increased to 14 mm (corresponding to the steel ratio of 0.122), the *SI* coefficient varied at similar levels to those of the 10 mm-thick steel-plate group.

### 4.2. Design Formulas

According to the research by Ding et al. [27] on the axial-strength calculations of the CFST columns, the axial-bearing capacity of CFST columns can be expressed as the sum strength of the steel tube and the infilled concrete. The strength elevation that was induced from the composition between the steel tube and infilled concrete is mainly presented as the strength increase in infilled concrete. Then, the axial compressive strength of the infilled concrete was multiplied with a strengthening coefficient as follows.

**Figure 14:**
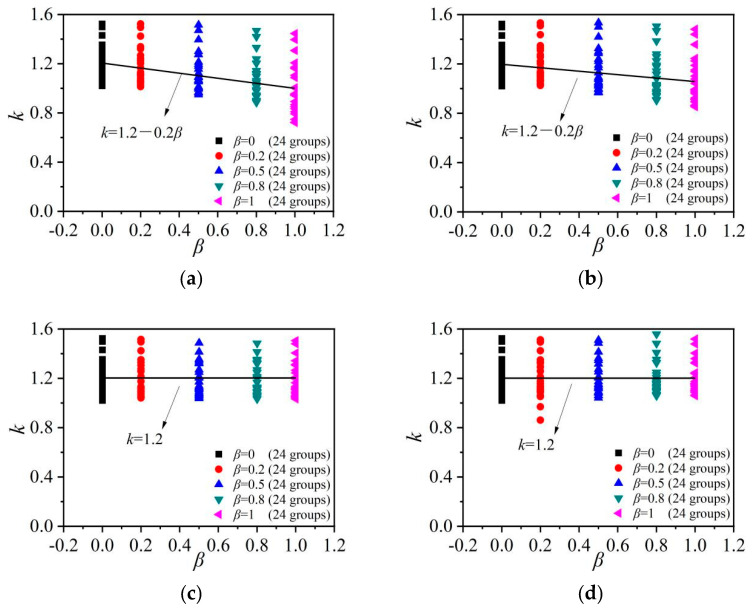
Calibrations of the relations between *k* and *β* in the notched columns. (**a**) Horizontal notch on the side plate; (**b**) Horizontal notch at the corner; (**c**) Vertical notch on the side plate; (**d**) Vertical notch at the corner.

*N*_u_ = *f*_c_*A*_c_ + 1.2*f*_y_*A*_s_ (4)

where 1.2 is the shape constraint coefficient of square steel tubes. For the columns with a notch in the steel tube, the confinement to the core concrete decreased, and then the shape constraint coefficient of the steel tube was also reduced. The axial-bearing capacity of the notched examples could be assumed as follows:

*N*_uc_ = *f*_c_*A*_c_ + *kf*_y_*A*_s_ (5)

where *k* is the shape constraint coefficient of the notched square steel tubes. As indicated in Figure 13, *β* was the most important parameter and imposed a significant impact on *k*. The relations between *k* and *β* for 408 columns are shown in Figure 14. For the vertically notched columns, there was no significant influence of *β* on the axial-bearing capacity, and then *k* of the vertically notched columns remained 1.2, while *k* for the horizontally notched columns could be taken as:

*k* = 1.2 − 0.2*β* (6)

By substituting Equation (6) into Equation (5), the axial capacity formula of the horizontally notched CFST columns could be obtained as follows:

*N*_uc_ = *f*_c_*A*_c_ + (1.2 − 0.2*β*) *f*_y_*A*_s_ (7)

**Figure 15:**
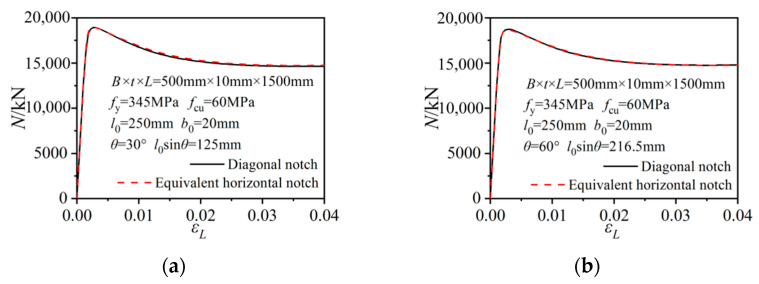
Comparisons of the load–axial strain curves between the diagonally notched columns and the equivalent horizontal-notched columns. (**a**) *θ* = 30°; (**b**) *θ* = 60°.

The horizontal and vertical notches were special cases of diagonal notches. The diagonal notch can be described with a *l*_0_sin*θ* long horizontal notch and a *l*_0_cos*θ* long vertical notch. Given that the axial strength reduction mainly occurred in the horizontally notched columns, the effect of the diagonal notch on column strength can be calculated based on the horizontal notch component. Figure 15 gives the axial-load versus axial-column-strain relations of the selected diagonally notched columns and the columns with only the equivalent horizontal notch component. The initial stiffnesses, strength evolutions and later degradation processes all agreed well with each other. Therefore, the calculation formula Equation (7) can be further extended to the diagonally notched columns in a more universal form as follows:

*N*_uc_ = *f*_c_*A*_c_ + (1.2 − 0.2*β*sin*θ*) *f*_y_*A*_s_ (8)

### 4.3. Calculation Formula Verification

Table 1 summarizes the shape confinement coefficients of the analyzed notched columns. And Equations (9) and (10) give the available axial-strength calculation equations of the notched CFST columns in references [27] and [14], respectively. Comparisons of the theoretical values from the proposed equations, the available reference equations, and the test data are given in Table 2. The mean ratios of the test data (*N*_uc,t_) to the analytical results calculated by Equation (8) (*N*_uc,Equation(8)_), Equation (9) (*N*_uc,Equation(9)_), and Equation (10) (*N*_uc,Equation(10)_) were 0.996, 1.025, and 1.118, respectively, with the corresponding dispersion coefficients being 0.078, 0.067, and 0.099.

(9) $$N_{\mathrm{uc}}=f_{c}A_{c}[1+(1.2-0.69\beta_{1}-0.81\beta_{2})\Phi ]\;\beta_{1}=\{\begin{array}{cc} l_{0}/4B & \mathrm{Horizontal}\;\mathrm{notch}\\ b_{0}/4B & \mathrm{Vertical}\;\mathrm{notch} \end{array}\;\;\beta_{2}=\{\begin{array}{cc} b_{0}/4B & \mathrm{Horizontal}\;\mathrm{notch}\\ l_{0}/4B & \mathrm{Vertical}\;\mathrm{notch} \end{array}$$

*N*_uc_ = *f*_c_*A*_c_ + *f*_y_ [4*t* (*B* − *t*) − (*l*_0_sin*θ* + *b*_0_cos*θ*) *t*] (10)

**Figure 16:**
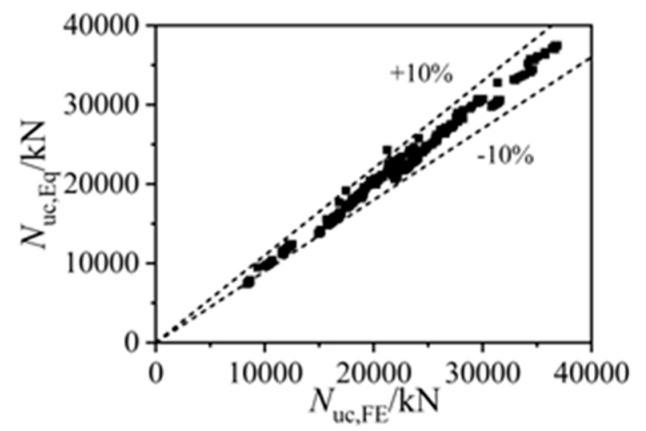
Comparisons of the finite element and design formula results.

Figure 16 gives the comparisons of 408 FE results in the axial strength and the corresponding analytical strengths by Equation (8), and Table 3 lists the comparisons of different analytical methods. The average ratio of the FE strengths to the analytical results was 1.017, and the dispersion coefficient was 0.036, while the ratios of the FE-simulated strengths (*N*_uc,FE_) to the analytical results by Equation (9) (*N*_uc,Equation(9)_) and Equation (10) (*N*_uc,Equation(10)_) were 1.022 and 1.102, respectively, with the corresponding dispersion coefficients being 0.039 and 0.041. The precise axial-strength predictions by Equation (8) indicated the applicability of the proposed methods on predicting the bearing strength of square CFST columns with various notch types.

## 5. Conclusions

In this paper, the working mechanisms and the composite effect in the notched square CFST columns were investigated. The refined 3-D FE models were established and the effectiveness was validated by the experimental results. A parametric study was performed to explore the effect of notch configurations on the axial strength and mechanical behaviors of the rectangular CFST columns. With the aid of the FE results, the practical axial-strength calculation equations, considering the composite actions in the square CFST columns, were proposed. The effectiveness of the proposed formulas was verified through the experimental and FE results.

The FE models adopted the advanced concrete constitutive model, material parameters, and detailed contact interactions. Stress and deformation conditions in the steel tube and the core concrete, and the composite working mechanisms between two components, were all captured.

Horizontal, vertical, and diagonal notches had different influences on the bearing modes in the core concrete, the axial-column-strength developments, and the composite effect. The horizontal notch could reduce the load participation ratio of the steel tube. Moreover, stress states of the core concrete changed from the axially loaded mode to the eccentric compression mode with the increase in notch lengths, leading to reductions in the axial column strengths. The vertical notch presented little effect on the axial column strength. The tube plates around the notch buckled under excessive axial compressive deformation during the later strength degradation stages. The influence of diagonal notches lay between those of the horizontal and vertical notches, and the resulting effect was similar to horizontally notched columns with the notch length as the horizontal projection length of the diagonal notch.

Based on the parametric study and the regression analysis, the ultimate axial-strength calculation formula of the notched rectangular CFST columns was proposed. The confinement effect of the notched square steel tubes was described with a coefficient of (1.2 − 0.2*β*sin*θ*), which could take into consideration the influence of notch inclination angles and lengths. The formula had a simple form, and showed good agreement with the test data and numerical results.

## Figures and Tables

**Table 1:** Shape confinement coefficient *k* values for different notch modes.

Section Form	Notched Mode	Formula Form	The Value of *k*
Square	Horizontal notch	*N*_uc_ = *f*_c_*A*_c_ + *kf*_y_*A*_s_	1.2 − 0.2*β*
	Vertical notch		1.2
	Diagonal notch		1.2 − 0.2*β*sin*θ*

**Table 2:** Comparisons between the test data, FE results, and analytical predictions.

Specimen	*N*_uc,t_ (kN)	*N*_uc,FE_ (kN)	*N*_uc,t_/*N*_u,FE_	Equation (8)		Equation (9)		Equation (10)	
				*N*_uc,Eq_ (kN)	*N*_uc,t_/*N*_uc,Eq_	*N*_uc,Eq_ (kN)	*N*_uc,t_/*N*_uc,Eq_	*N*_uc,Eq_ (kN)	*N*_uc,t_/*N*_uc,Eq_
SCN-1	2049	2013	1.018	2022	1.014	2005	1.022	1800	1.138
SCN-2	1850	1852	0.999	1924	0.961	1904	0.971	1675	1.104
SCN-3	2040	2006	1.017	2022	1.009	2005	1.017	1800	1.133
SCN-4	1970	1927	1.023	1940	1.015	1920	1.026	1690	1.166
SLN-5	2075	2065	1.005	2135	0.972	2086	0.995	1927	1.077
SLN-6	2016	1997	1.010	2119	0.951	2031	0.993	1912	1.055
SLN-7	1942	1925	1.009	2119	0.916	1953	0.994	1912	1.016
SLN-8	2064	2071	0.997	2119	0.974	2070	0.997	1912	1.080
SLN-9	1980	1999	0.991	2135	0.927	2047	0.967	1927	1.028
SLN-10	1930	1938	0.996	2135	0.904	1969	0.980	1927	1.002
SFT-11	2090	2067	1.011	2119	0.986	2119	0.986	1924	1.086
CFSST-S-1	966	959	1.007	813	1.188	808	1.196	722	1.338
CFSST-S-2	899	893	1.007	797	1.128	791	1.137	701	1.282
Average values			1.007		0.996		1.022		1.116
Dispersion coefficient			0.009		0.078		0.065		0.096

**Table 3:** Comparisons of different analytical methods.

*N*_uc,FE_/*N*_uc,Eq_	Equation (8)	Equation (9)	Equation (10)
Average value	1.017	1.022	1.102
Dispersion coefficient	0.036	0.039	0.041

## Data Availability

The data presented in this study are available on request from the corresponding author. The data are not publicly available as the data forms part of an ongoing study.
